# Febrile Seizures among Children Admitted to the Department of Paediatrics of a Tertiary Care Centre: A Descriptive Cross-sectional Study

**DOI:** 10.31729/jnma.7197

**Published:** 2022-04-30

**Authors:** Sumita Poudel, Sudhir Adhikari, Rohit Thapa, Biraj Parajuli, Shanti Regmi, Prajjwal Kunwar

**Affiliations:** 1 Department of Paediatrics, Chitwan Medical College Teaching Hospital, Bharatpur, Chitwan, Nepal; 2 Chitwan Medical College Teaching Hospital, Bharatpur, Chitwan, Nepal; 3 Kathmandu Medical College and Teaching Hospital, Sinamangal, Kathmandu, Nepal

**Keywords:** *febrile seizure*, *hyponatremia*, *recurrence*

## Abstract

**Introduction:**

Febrile seizure is the most common convulsive event in children younger than 60 months. Fever plays an important role in causing disturbances in fluid and electrolyte balance, also hyponatremia has been thought to enhance the susceptibility to seizures. The objective of this study is to find out the prevalence of febrile seizures among children admitted to the Department of Paediatrics of a tertiary care centre.

**Methods:**

A descriptive cross-sectional study among children admitted to the Department of Paediatrics was done at a tertiary care centre between December, 2020 to September, 2021. Ethical approval was taken from the Institutional Review Committee (Reference number: 077/078-098). A total of 1052 children were included in this study. A convenience sampling technique was used. Statistical Package for the Social Sciences version 25.0 was used for data analysis. Point estimate at 95% Confidence Interval was calculated along with frequency and proportion for binary data.

**Results:**

Among 1052 children, the prevalence of febrile seizure was 100 (9.50%) (7.73-11.27 at 95% Confidence Interval). Among these 100 patients, 68 (68%) had simple febrile seizures while 32 (32%) had recurrent febrile seizures.

**Conclusions:**

The prevalence of febrile seizures was found to be higher than in other studies conducted in similar settings. This knowledge may be of practical value in advising parents or caregivers of the risk of a febrile seizure and its recurrence.

## INTRODUCTION

Febrile seizures (FS) are the most common form of childhood seizures.^1^ American Academy of Paediatrics (AAP) defines a febrile seizure as a seizure occurring in a febrile child between 6 to 60 months without intracranial infection, metabolic disturbance, or history of afebrile seizures.^2^

About 2-5% of children are estimated to undergo at least one seizure during a febrile illness before the age of 5 years accounting for 30% of all seizures among children.^3^ It was suggested that variations in serum electrolyte levels will increase the susceptibility to seizures and recurrence of FS during childhood. Low sodium levels can lead to depolarization of nerve cells and precipitate convulsions.^4^ Though AAP does not recommend serum electrolytes be obtained routinely in children with first febrile seizure various studies show hyponatremia as a risk factor for a recurrent febrile seizure.^5,6^

The objective of this study is to find out the prevalence of febrile seizures among children admitted to the Department of Paediatrics of a tertiary care centre.

## METHODS

A descriptive cross-sectional study was conducted in the Department of Paediatrics of Chitwan Medical College Teaching Hospital (CMC-TH) from December, 2020 to September, 2021. Children aged 6 months to 5 years were included in the study. The study was approved by the Institutional Review Committee (IRC) of Chitwan Medical College (Reference number: CMC-IRC/077/078-098). Written informed consent was obtained from the parents. The children aged between 6 and 60 months with a normal neurological examination at admission were included in the study. Similarly, children with signs of central nervous system infections, children with gastroenteritis with dehydration, children with developmental delay, children with neurometabolic disorders, children with a history of afebrile seizure, children who were given intravenous fluids or diuretics, children with malabsorption syndrome or severe acute malnutrition were excluded from the study.

Sample size was calculated using the formula:

n = (Z^2^ × p × q) / e^2^

  = (1.96^2^ × 0.0461 × 0.9539) / 0.02^2^

  = 423

Where,

n = minimum required sample size
Z = 1.96 at 95% Confidence Interval (CI)
p = prevalence of febrile seizure, 4.61%^7^
q = 1-p
e = margin of error, 2%

The minimum required sample size was 423, but after doubling, a sample size of 846 was attained. However, as convenience sampling was done, a sample of 1052 was taken for the study.

Simple febrile seizures were defined as a single episode of febrile seizure in children after ruling out central nervous system infection or any other defined cause of the seizure. Recurrent febrile seizures were defined as recurrent febrile seizures in children within 24 hours of admission. The demographic characteristics such as sex, age, body temperature, convulsion time, seizure frequency, family history of seizures, history of previous seizures, underlying diseases (upper respiratory tract infections, otitis media, lower respiratory tract infection, upper and lower urinary tract infection, gastroenteritis, etc.) and serum electrolyte status were documented for each patient. In all patients, the sodium, and potassium levels were determined at the beginning of admission. A serum sodium level of less than 135 meq/l was taken as a cut-off value to label as hyponatremia. Serum sodium measurement between 130-135 meq/ml was considered mild or Relative Hyponatremia (RH). Patients were monitored after hospitalisation within 24 hours for seizure recurrence. The data were collected in a proforma.

Data were entered in Microsoft Excel and analysed using Statistical Package for the Social Sciences (SPSS) version 25.0. The point estimate was done at 95% Confidence Interval and descriptive statistics which included frequency and percentage were derived and presented in tables and figures.

## RESULTS

**Figure 1:**
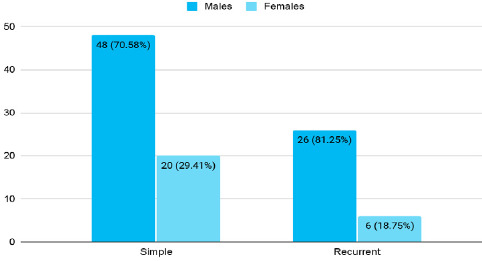
Distribution of febrile convulsion according to gender (n= 100).

Among 1052 children, the prevalence of febrile seizure was 100 (9.50%) (7.73-11.27 at 95% Confidence Interval). Among these 100 patients, 68 (68%) had simple febrile seizures while 32 (32%) had recurrent febrile seizures. The mean age of simple febrile seizure was 27.21±13.36 months consisting of 48 (70.58%) males and 20 (29.41%) females. The mean age of recurrent febrile seizure was 26.91±17.10 months consisting of 26 (81.25%) males and 6 (18.75%) females (Figure 1).

**Figure 2:**
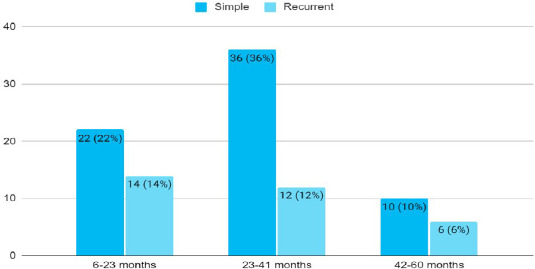
Distribution of febrile convulsion according to age group (n= 100).

Among 100 children with febrile seizures, febrile convulsion was more common in 23-41 months (Figure 2).

Upper Respiratory Tract Infection (URTI) was present in most of the children with febrile convulsion 44 (44%) being the most common focus of infection (Table 1).

**Table 1:** Distribution of febrile convulsion according to the focus of infection (n= 100).

Focus of infection	Simple febrile seizure n(%)	Recurrent febrile seizure n(%)
Lower Respiratory Tract Infection (LRTI)	17 (17)	13 (13)
Upper Respiratory Tract Infection (URTI)	32 (32)	12 (12)
Urinary Tract Infection (UTI)	8 (8)	1 (1)
Acute Gastroenteritis (AGE)	4 (4)	6 (6)
Others	7 (7)	-
Total	68 (68)	32 (32)

In patients with simple febrile seizure, the mean value of sodium was 136.79±3.02 and potassium was 4.35±0.49. Similarly, in patients with recurrent febrile seizure, the mean values of sodium and potassium were 134.84±2.89 and 4.50±0.53 respectively.

Among 100 patients with febrile seizure, the prevalence of overall hyponatremia was found to be 36 (36%). Out of these 36 patients, 18 (50%) had simple febrile seizures and 18 (50%) had recurrent febrile seizures within 24 hours. Among 64 (64%) children with serum sodium level >135 meq/l, 13 (20.3%) developed recurrence within 24 hours (Table 2).

**Table 2:** Serum sodium level, and mean seizure episodes (n= 100).

Serum sodium level	n (%)	Seizure episodes (Mean±SD)
Relative hyponatremia (130-134)	36 (36)	1.94±1.12
Normal (≥135)	64 (64)	1.28±0.60

Also, mean levels of haemoglobin and random blood sugar in the patients with simple febrile seizure was 11±1.15 (n= 68) and 104.58±26.85 (n= 32) respectively. Similarly, the mean levels of haemoglobin and random blood sugar in the patients with recurrent febrile seizure was 11.24±0.94 (n= 43) and 110.50±41.05 (n= 24) respectively.

## DISCUSSION

Febrile convulsion is a terrifying event for the parents and requires emergent medical attention. Attempts have been made to identify predisposing risk factors and predictors of seizure recurrence. This provides information on the need for admission of the child and the importance of counselling parents about seizure recurrence.^8^

A study conducted in a tertiary care centre in Western Nepal reported the prevalence of febrile seizure in children to be 4.61% which was lower when compared to our study.^7^ A study conducted in India found hyponatremia in 37.5% of the total febrile seizure cases similar to our study.^9^ A study conducted in Karnataka among cases of febrile seizure found similar results.^10^ Similar studies also found a higher incidence of hyponatremia among febrile seizure cases.^11,12^ Prevalence of recurrent febrile seizure is also high in another similar study comparable to our study.^13^ However, lower levels of hyponatremia in such patients have been reported in contrast to our study.^4^

Studies have found that there is a significant association between recurrent febrile convulsion and lower level of serum sodium in which serum sodium concentration is lowest in those patients with recurrent febrile convulsion.^13^ There are studies stating that the measurements of serum sodium levels and hyponatremia diagnosis have a key role in predicting FS occurrence and recurrence. A significant association between recurrent febrile convulsion and a lower level of serum sodium was observed in the study.^14^ Studies have shown that serum sodium levels are valuable in children with febrile convulsion showing a higher probability of a repeat convulsion with a decrease in serum sodium levels, possibly within the same febrile period.^6,12^ However, studies have been reported in contrast to this evidence,^15,16^ and the role of relative hyponatremia instead of serum sodium levels in the predisposition of the child to simple febrile seizure has been speculated.^17,18^

Lower levels of mean sodium levels have been reported in patients with repeat convulsions, which was comparable to our study.^6^ Lower sodium levels in children with complicated convulsions compared to those having simple convulsions have also been reported.^19^ In contrast to our study, a showed that the mean serum sodium for febrile seizures in 24 hours (135.48 mmol/l) did not differ from those with simple febrile seizures (135.56 mmol/l).^20^ Male gender predominance was almost well documented in other studies similar to our study.^12,21^ The maximum age group of children was 23-41 months in our study similar to another study.^13^ Mean age of presentation of febrile seizures is lower than our study have been reported.^22^

The key limitations of our study include the descriptive nature of the study which doesnot allow the measurement of the association between the variables including febrile seizure, serum sodium levels, and the recurrence of febrile seizures could not be made and no sort of causality could be established. A larger sample size and multi-centre study could increase the generalizability of the findings of this study.

## CONCLUSIONS

The prevalence of febrile seizures was found to be higher than in other studies conducted in similar settings. This knowledge may be of practical value in advising parents or caregivers of the risk of a febrile seizure and its recurrence. Hyponatremia was prevalent in the patients which could indicate the need for more research regarding serum sodium levels and febrile seizures.
